# Tandem mass tag-based proteomics analysis reveals the multitarget mechanisms of *Phyllanthus emblica* against liver fibrosis

**DOI:** 10.3389/fphar.2022.989995

**Published:** 2022-10-13

**Authors:** Puyang Gong, Kehuan Yin, Xiaomin Luo, Jian Gu, Rui Tan, Yan Wu, Dapeng Li

**Affiliations:** ^1^ College of Pharmacy, Southwest Minzu University, Chengdu, China; ^2^ College of Life Science and Engineering, Southwest Jiaotong University, Chengdu, China; ^3^ College of Pharmacy, Shenzhen Technology University, Shenzhen, China; ^4^ West China School of Pharmacy, Sichuan University, Chengdu, China

**Keywords:** *Phyllanthus emblica*, liver fibrosis, TMT-based proteomics, multi-target mechanism, differentially expressed proteins

## Abstract

*Phyllanthus emblica* (PE), a traditional multiethnic herbal medicine, is commonly applied to treat liver diseases. Our previous study demonstrated that aqueous extract of PE (AEPE) could alleviate carbon tetrachloride (CCl_4_)-induced liver fibrosis *in vivo*, but the underlying molecular mechanisms are still unclear. The present study was undertaken to clarify the multitarget mechanisms of PE in treating liver fibrosis by proteomics clues. A CCl_4_-induced liver fibrosis rat model was established. The anti-liver fibrosis effects of chemical fractions from AEPE were evaluated by serum biochemical indicators and pathological staining. Additionally, tandem mass tag (TMT) - based quantitative proteomics technology was used to detect the hepatic differentially expressed proteins (DEPs). The Kyoto Encyclopedia of Genes and Genomes (KEGG) pathway enrichment, gene ontology (GO) enrichment and protein-protein interaction (PPI) network were used to perform bioinformatics analysis of DEPs. Western blot analysis was used to verify the key potential targets regulated by the effective fraction of AEPE. The low-molecular-weight fraction of AEPE (LWPE) was determined to be the optimal anti-liver fibrosis active fraction, that could significantly improve ALT, AST, HA, Col IV, PCIII, LN, Hyp levels and reduce the pathological fibrotic lesion of liver tissue in model rats. A total of 195 DEPs were screened after LWPE intervention. GO analysis showed that the DEPs were related mostly to extracellular matrix organization, actin binding, and extracellular exosomes. KEGG pathway analysis showed that DEPs are mainly related to ECM-receptor interactions, focal adhesion and PI3K-Akt signaling pathway. Combined with the GO, KEGG and Western blot results, COL1A2, ITGAV, TLR2, ACE, and PDGFRB may be potential targets for PE treatment of liver fibrosis. In conclusion, LWPE exerts therapeutic effects through multiple pathways and multiple targets regulation in the treatment of liver fibrosis. This study may provide proteomics clues for the continuation of research on liver fibrosis treatment with PE.

## 1 Introduction

Hepatic fibrosis, as a crucial pathological feature, exists in almost all chronic liver diseases triggered by hepatitis virus, alcoholism, cholestasis and lipodystrophy, etc (Shang et al., 2022). Without appropriate therapy, hepatic fibrosis could further deteriorate into cirrhosis, portal hypertension and hepatocellular carcinoma (Anuja et al., 2018). Hepatic fibrosis threatens nearly 2% of the worldwide population and leads to more than one million deaths each year (Qiao et al., 2020). To date, there are still no available drugs approved for the clinical treatment of liver fibrosis (Shi et al., 2020). The development of liver fibrosis comprises mainly inflammation, oxidative stress, hepatic stellate cell (HSC) activation, extracellular matrix (ECM) deposition and formation of fibrillar scar matrix (Qiao et al., 2020). In view of the complicated and dynamic physiopathological mechanism of liver fibrosis, the development of comprehensive medications is urgently needed. Fortunately, traditional Chinese medicine (TCM) has advantages in the treatment of liver fibrosis due to its multiple components and multiple targets (Shan et al., 2019).

*Phyllanthus emblica* L. (PE), an edible and medicinal dual-purpose plant, belongs to the Euphorbiaceae family which is widely distributed in China, India, Nepal and Malaysia (Muthuraman et al., 2011; Sripanidkulchai and Junlatat, 2014). The fruits of PE first used as a traditional Chinese medicinal material were documented in ‘*Xin xiu ben cao*’ (Newly Revised Materia Medica) in the Tang Dynasty (Huang et al., 2021). PE possesses the traditional efficacies of clearing heat and cooling blood, eliminating food and invigorating the stomach, generating body fluid and relieving cough, and used for treating blood heat, blood stasis and indigestion (Wang et al., 2019). Blood heat and stasis are the main pathogenesis of liver fibrosis according to the theory of TCM (Zhao et al., 2022). Hence, the traditional efficacies of PE are consistent with the basic principle of TCM for the treatment of liver fibrosis. PE was consisted in many herbal medicine compounds for treating the hepatic fibrosis such as *Fuzheng Rougan* formulae (Liu et al., 2014). Furthermore, Tibetans, Uygurs, Mongolians and other ethnic groups in China also use PE to treat blood fever and hepatobiliary diseases (Zhu et al., 2015).

Many modern pharmacological studies have substantiated that PE extract and its active compounds exhibit excellent hepatoprotective effects on various biological models of liver diseases (Yin et al., 2022). Previous studies have shown that PE extract could inhibit the activation of HSC-T6 cells induced by leptin *in vitro* (Lu et al., 2016), and reverse the pathological changes of early liver fibrosis induced by carbon tetrachloride (CCl_4_) and thioacetamide *in vivo* (Tasduq et al., 2005; Mir et al., 2007). Our previous study have also demonstrated that the aqueous extract of PE (AEPE) could alleviate CCl_4_ induced liver fibrosis through antioxidant and anti-inflammatory effects, inhibiting stellate cell activation and reducing the extracellular matrix (Yin et al., 2021). These evidences suggested that PE may be a potential source of multitarget anti-hepatic fibrosis combination drugs. Importantly, the multitarget mechanism of PE against hepatic fibrosis should be elucidated first.

A great number of protein molecules will be changed in regard to quantity and quality in the development of liver fibrosis (Zardi et al., 2008). Thus, high throughput proteomics combined with bioinformatics for data mining provides a preferable method for large-scale screening and identification of differential proteins for disease, and to predict targets and mechanisms of drugs (Yuan et al., 2020). Tandem mass tag (TMT) - based proteomics is an *in vitro* peptide labelling quantitative technique, that can react with amino labelling, and simultaneously achieve the qualitative and quantitative characterization of multiple sample proteomics through high-precision mass spectrometry analysis. Due to the high repeatability and high sensitivity of the method, it has become one of the most popular proteomic methods for finding biomarkers and screening disease targets (Lan et al., 2021; Yu et al., 2021).

**FIGURE 1:**
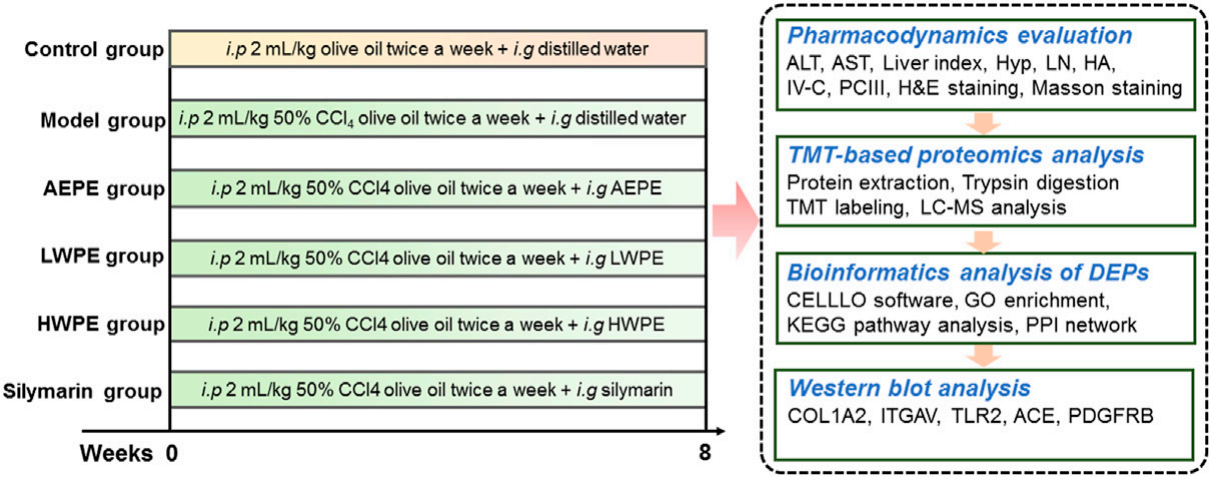
Overview of the experimental design of proteomics.

In this study, a rat model of liver fibrosis was established by injection with CCl_4_, and the active chemical fraction of AEPE was determined. Further, differentially expressed proteins (DEPs) in liver tissue were analyzed by TMT-based quantitative proteomics. The key targets and underlying mechanisms of regulation by the active fraction of AEPE were explored by bioinformatics analysis. In addition, the key target proteins were verified by Western blotting (Figure 1). The results provide more ideas for the mechanism and targets of PE in the treatment of liver fibrosis.

## 2 Materials and methods

### 2.1 Chemicals and reagents

Olive oil was obtained from Sigma-Aldrich (St. Louis, MO, United States). CCl_4_ was purchased from Shanghai Macklin Biochemical Co., Ltd (Shanghai, China). Silymarin was bought from Madaus (Cologne, Germany). Biochemical assay kits for alanine aminotransferase (ALT), aspartate aminotransferase (AST) and Hydroxyprolin (Hyp) were provided by Institute of Jiancheng Bioengineering (Nanjing, China). Enzyme-linked Immunosorbent Assay (ELISA) and BCA protein quantification kits were obtained by Shanghai Enzyme Link Biotechnology Co., Ltd (Shanghai, China). Rabbit anti-rat polyclonal antibody glyceraldehyde-3-phosphate dehydrogenase (GAPDH), integrin subunit alpha V (ITGAV), platelet derived growth factor receptor beta (PDGFRB), toll-like receptor 2 (TLR2) were purchased from Affinity Biosciences (Changzhou, China). Rabbit anti-rat polyclonal antibody collagen type I alpha 2 chain (COL1A2) and angiotensin I converting enzyme (ACE) were purchased from Beijing Biosynthetic Biotechnology Co., Ltd (Beijing, China) and Wuhan Service biotechnology Co., Ltd (Wuhan, China), respectively. Goat anti-rabbit fluorescently-labeled secondary antibody was purchased from Multi sciences biotechnology Co., Ltd (Shanghai, China).

### 2.2 Preparation of test drugs

The AEPE and its fractions samples were prepared using the same batch of dried PE fruit (voucher specimens no. 20201212) as in our previous study (Yin et al., 2021; Luo et al., 2022). Briefly, 2000 g of PE was powdered and immersed in distilled water on a rotary shaker for 24 h at a mass ratio of 1:10 at 37°C. Then, the extract was filtered and concentrated to 2000 ml. The 1000 ml extract was precipitated by adding 4 volumes of anhydrous ethanol. The supernatant was concentrated to 1 g (crude drug)/ml to obtain the low-molecular-weight fraction of AEPE (termed LWPE). The resulting precipitate was lyophilized and the high-molecular weight fraction of AEPE (termed HWPE) was obtained. In our previous studies, the main chemical ingredients in AEPE were quantified by high-performance liquid chromatography (HPLC) and the content of crude polysaccharides in HWPE was determined by phenol-sulfuric acid method (Yin et al., 2021; Luo et al., 2022).

### 2.3 Quantitative analysis of low-molecular-weight fraction of AEPE by high-performance liquid chromatography

The contents of gallic acid and methyl gallate were detected by an Agilent 1260 liquid chromatograph system. The chromatographic separation was performed on a Kromasil 100-5-C_18_ (4.6 × 150mm) column at 37°C.The mobile phase consisted of water containing 0.1% formic acid (solvent A) and acetonitrile (solvent B). The linear gradient elution was as follows: 0–5 min, 3% B; 5–10 min, 3%–4% B; 10–20 min, 4%–12% B; 20–35 min, 12% B; 35–38 min, 12%–18% B; 38–55 min, 18%–40% B. The injection volume was 5 μl. The flow rate was 0.6 ml/min and the wave length was set at 254 nm.

### 2.4 Animals treatment

Male Wistar rats (180–220 g) were obtained from SPF (Beijing) Biotechnology Co., Ltd (Beijing, China). The animals received food and water allodially and were housed five per cage under well-controlled conditions (12 h light-dark cycle, room temperature 22.0 ± 2°C and room relative humidity 50%–60%) for 1 week for environment adaption. All experimental procedures were audited and approved by the animal ethics committee of Southwest Minzu University (No. 2021-22).

The rats were randomized into the following six groups (10 rats in each group): the control group, model group, AEPE group, LWPE group, HWPE group and silymarin group. The control group received intraperitoneally injected olive oil at a dose of 2 ml/kg twice each week for eight successive weeks, while the rats in the other groups received 2 ml/kg 50% CCl_4_ olive oil. The silymarin group was gavaged daily with 42 mg/kg silymarin aqueous solution. AEPE, LWPE and HWPE aqueous solutions were administered intragastrically to the rats. The dosages of AEPE, LWPE and HWPE were equivalent to their contents in 1.8 g (crude herb)/kg, which was the optimal dose obtained from our previous experiments (Yin et al., 2021). At the end of the 8th week, all rats were anaesthetized with ether, and blood and liver samples were collected for subsequent examination.

### 2.5 Serum biochemical analyses

The levels of ALT and AST in serum were analyzed by commercially available kits according to the manufacturer’s instructions.

### 2.6 Detection of liver fibrosis biomarkers and hydroxyproline contents

Serum content of HA, LN, IV-C, and PCIII was measured by ELISA. The level of Hyp in each liver tissue was analyzed with the alkaline hydrolysis method by commercial kit.

### 2.7 Liver histological examination

The liver tissues were fixed with 4% paraformaldehyde, dehydrated, embedded in paraffin, and 4-μm-thick sections were stained with hematoxylin-eosin (H&E) for histopathological evaluation. The severity of fibrosis was evaluated according to the criteria of Liu et al. (2006). Furthermore, Masson staining was applied to observe the changes of collagen deposition in liver tissue.

### 2.8 Tandem mass tag quantitative proteomics

#### 2.8.1 Protein extraction

Nine liver tissue samples (3 samples per group) collected from the control group, model group, and LWPE group were powdered in liquid nitrogen. The samples were mixed with SDT buffer, then homogenized using an MP homogenizer (24 × 2, 6.0 M/S, 60 s, twice). The homogenate was sonicated by a high-intensity ultrasonic processor and further boiled for 15 min. Next, the samples were centrifuged at 14,000 *g* for 40 min (Wang et al., 2020). The supernatant was collected and filtered with 0.22 µm filters, and the protein concentration of the filtrates was detected by a BCA kit (Zhu et al., 2014).

#### 2.8.2 Trypsin digestion and tandem mass tag labeling

The experimental procedure of this section referred to the literature of Wisniewski et al. (2009). For each sample, 0.2 mg of protein was mixed with 30 μl SDT buffer. Ultrafiltration (Microcon units, 10 kD) was performed to remove the DTT, detergent and other small molecule ingredients using UA buffer (8 M urea, 150 mM Tris-HCl pH 8.0). Then, 100 μL iodoacetamide (100 mM IAA in UA buffer) was added to the sample to intercept reduced cysteine residues, and the sample was incubated in darkness for 0.5 h. The filters were washed three times with 100 μl UA buffer and 100 μl 100 mM TEAB buffer twice. Ultimately, the protein suspensions were digested with 4 μg trypsin in 40 μl TEAB (1:10) buffer at 37°C overnight. According to the calculation of the frequency of tryptophan and tyrosine in vertebrate proteins, the peptide content was estimated by UV spectral density at 280 nm with an extinction coefficient of 1.1 in a 0.1% (g/L) solution. Then, 100 μg of peptide mixture from each sample was labelled according to the instructions of a commercial TMT kit (Thermo Fisher Scientific, United States).

#### 2.8.4 High pH reversed-phase fractionation and LC-MS analysis

TMT-labelled peptides were fractionated into 10 fractions through an incremental acetonitrile step-gradient elution according to the instructions of the Pierce high pH reversed-phase fractionation kit (Thermo scientific, United States). Each fraction was injected for nano liquid chromatography tandem mass spectrometry (LC-MS/MS) analysis performed on an EASY nLC and Q Exactive mass spectrometer (Thermo scientific, United States). Formic acid in 0.1% aqueous solution was used as buffer A, and 0.1% formic acid acetonitrile aqueous solution (84% acetonitrile) was used as buffer B. The samples were separated on a trap column (Thermo, 100 μm × 2 cm) connected to a C_18_ reversed-phase analytical column (Thermo, 75 μm × 10 cm). The flow rate was 300 nl/min. The liquid phase gradient was set as follows: 0–55% buffer B for 80 min, 55–100% buffer B for 5 min, and 100% buffer B for 5 min. On-line mass spectrometry analysis was performed by a Q Exactive mass spectrometer in positive ion mode. The normalized collision energy was 30 eV. The scanning range was 300–1800 m*/z*. The automatic gain control target was set to 3e6, and the maximum injection time was set to 10 milliseconds. The dynamic exclusion time was 40.0 s.

#### 2.8.6 Database searching

The MASCOT engine version 2.2 embedded into Proteome Discoverer 1.4 software was applied for identification and quantitation analysis of MS raw data of samples. Ensembl_Rattus_29107_20200311. fasta was used as the database. Trypsin was specified as the cleavage enzyme, and the number of missed cleavage sites was 2. The mass error tolerance of the first-level search precursor ion was ±20 ppm; the mass error tolerance of the second-level fragment ion was 0.02 Da. The peptide false discovery rate (FDR) was adjusted to ≤0.01.

#### 2.8.7 Bioinformatics analysis

DEPs were subjected to multiple bioinformatic analyses. Gene Ontology (GO) annotation and Kyoto Encyclopedia of Genes and Genomes (KEGG) pathways enrichment analysis was performed using Database for Annotation, Visualization and Integrated Discovery (DAVID) Bioinformatics Resources (https://david.ncifcrf.gov). The potential protein-protein interactions (PPI) were analyzed *via* the STRING database (https://string-db.org) and CytoScape 3.9.0 software.

### 2.9 Western blot analysis

Frozen liver tissues were washed 3 times with phosphate buffered saline (PBS), cut into pieces and placed in 10 times the amount of frozen radioimmunoprecipitation assay (RIPA) lysis buffer to prepare tissue homogenates. Then, the protein concentration was determined with a BCA quantification kit. The proteins were separated on sodium dodecyl sulfate-polyacrylamide gel electrophoresis (SDS-PAGE) gel and then transferred to polyvinylidene fluoride membranes. The membrane was blocked with 5% skim milk (prepared with TBST) at room temperature for 60 min and then incubated overnight with rabbit monoclonal antibody (1:1000) at 4°C. After washing 3 times (5 min each time) with TBST, the membranes were incubated with goat antirabbit IgG (1:10000) for 1 h. Finally, enhanced chemiluminescence (ECL) solution was added to adjust the exposure conditions, and images were captured under the chemiluminescence imaging system.

### 2.10 Statistical analysis

Experimental data are presented as the mean ± standard error of the mean (S.E.M). The multiple comparisons were analyzed by one-way analysis of variance (ANOVA) followed by Dunnett’s post-hoc test by GraphPad Prism 9.0 software. A *p* value <0.05 was the significance threshold.

## 3 Results

### 3.1 Phytochemical investigation

**FIGURE 2:**
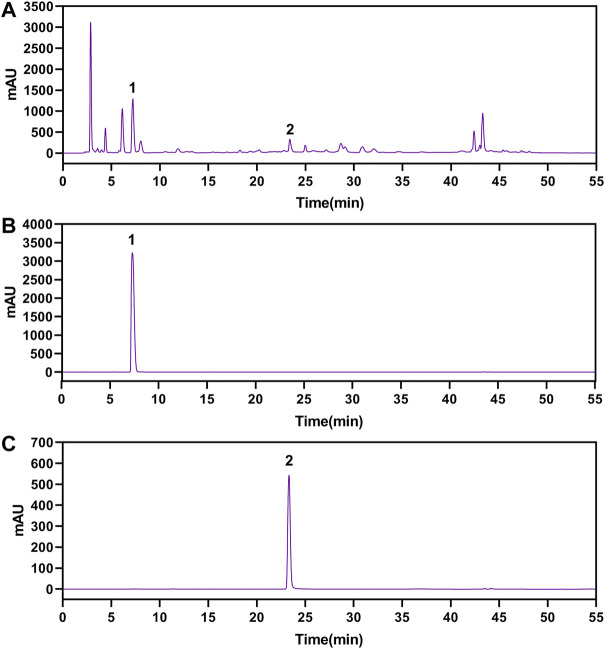
The HPLC chromatograms of LWPE **(A)**, gallic acid **(B)** and methyl gallate **(C)**.

The gallic acid and methyl gallate in LWPE were identified and quantified by HPLC, which presenting chromatographic peaks at retention times of 7.207 min for gallic acid and 23.297 min for methyl gallate (Figure 2). Quantifications were carried out by using six-point regression curves of gallic acid (y = 11921 + 828.68, *r* ^2^ = 0.9995) and methyl gallate (y = 12752 + 36.651, *r* ^2^ = 0.9999). The contents of gallic acid and methyl gallate in LWPE were 14.06 and 3.71 mg/ml, respectively.

### 3.2 Effects of aqueous extract of PE and its fractions on hepatic lesions in rats administered with carbon tetrachloride

**FIGURE 3:**
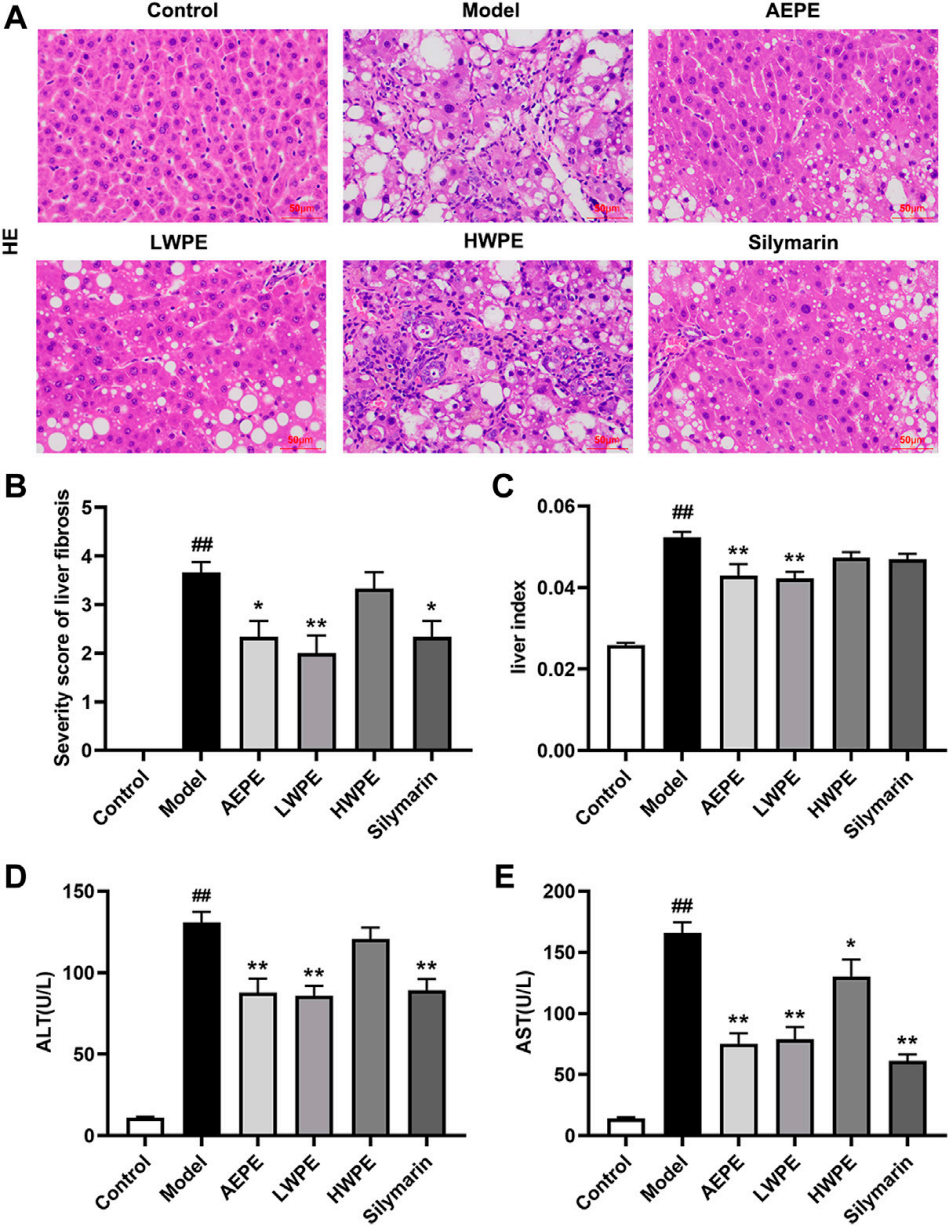
Effects of AEPE and its fractions on liver injury in rats administered with CCl_4_. **(A)** Representative histopathological sections of liver tissue from each group stained with H&E (magnification, ×200). **(B)** Severity score of hepatic fibrosis in H&E staining. **(C)** The liver-to-body weight ratio of each rat in the groups was calculated. **(D)** ALT levels in serum. **(E)** AST levels in serum. Control, control group. Model, model group. AEPE, aqueous extract of PE group. LWPE, low molecular weight fraction of PE group. HWPE, high molecular weight fraction of PE group. Silymarin, silymarin group. Data are expressed as the mean ± SEM (*n* = 7–10). ##*p* < 0.01 vs the control group; *p < 0.05, **p < 0.01 vs the model group.

H&E staining and serum biochemical analysis were performed to explore the protective effect of AEPE and its fractions on liver injury induced by CCl_4_. As shown in Figure 3A, liver tissues in the control group exhibited natural lobular architecture and cellular structure, and no appreciable pathological changes were observed. In contrast, the model group showed severe hepatocyte swelling and necrosis, lymphocyte infiltration, steatosis and fibrous septum. In the AEPE, LWPE and silymarin groups, the abnormal histological alterations were markedly reduced. Meanwhile, the average severity scores for liver fibrosis in rats treated with AEPE, LWPE and silymarin were significantly lowered compared to CCl_4_ controls (*p* < 0.05) (Figure 3B). However, HWPE showed no obvious influences on hepatic histological changes.

Additionally, after treatment with CCl_4_, the rat liver was congested and enlarged. As shown in Figure 3C, the increased liver index of the model rat was decreased after AEPE and LWPE treatment (*p* < 0.01). The measurement results of serum biochemical markers are shown in Figure 3D; and Figure 3E. The levels of serum ALT and AST in the model group were significantly higher than the levels of serum ALT and AST in the control group (*p* < 0.01), but decreased following AEPE, LWPE or silymarin treatment (*p* < 0.01). Nevertheless, significant variation of ALT levels was not observed in the HWPE group compared to the model rats.

### 3.3 Effects of aqueous extract of PE and its fractions on liver fibrosis in rats administered with carbon tetrachloride

**FIGURE 4:**
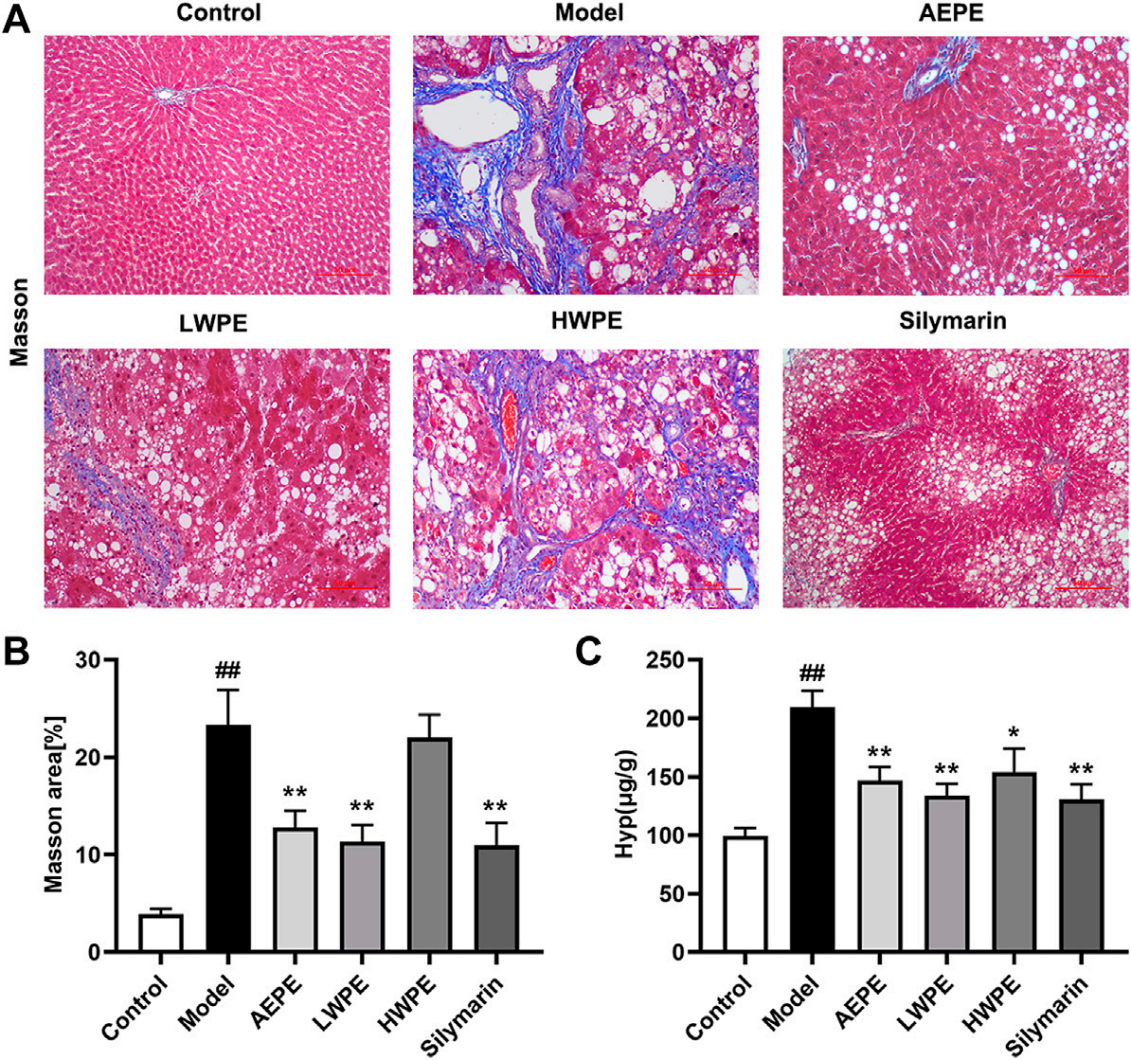
Effects of AEPE and its fractions on liver fibrosis in rats administered with CCl_4_. **(A)** Representative rat livers stained with Masson (magnification, ×200). **(B)** The positive regions of Masson staining. **(C)** Hyp contents in liver tissues. Data are expressed as the mean ± SEM (*n* = 7–10). ##*p* < 0.01 vs the control group; *p < 0.05, **p < 0.01 vs the model group.

Collagen is one of the main components of the ECM, which leads to the development of hepatic fibrosis (Zhang et al., 2020). Hence, collagen deposition in liver tissue was examined by Masson’s trichrome staining. As shown in Figure 4A, obvious collagen accumulation intersected at multiple portal areas in the model group, and pseudo lobule formation was also observed. Collagen deposition was reduced after treatment with AEPE, LWPE or silymarin. The fibrosis area of liver sections in the model group was significantly increased compared to the control group, but decreased in the test drug groups except the HWPE group (*p* < 0.01, Figure 4B). As a unique amino acid in collagen fibers, the Hyp content of liver tissue in the model group was significantly higher than that in the control group, but decreased in the AEPE, LWPE, HWPE and silyamrin groups (*p* < 0.05, Figure 4C).

Meanwhile, the serum biomarkers of fibrogenesis including LN, HA, IV-C, and PCIII were further detected by ELISA, and the results are displayed in Table 1. The levels of these indexes were significantly increased in rat serum after treatment with CCl_4_ (*p* < 0.01). Compared with the model group, the levels of LN, NA, PCIII, and IV-C in serum were decreased significantly after treatment with AEPE and LWPE (*p* < 0.05). However, there was no significant difference in the four indicators between the model rat and the HWPE group (*p* > 0.05).

**TABLE 1:** Effects AEPE and its fractions on the serum contents of LN, HA, IV-C and PCIII in CCl_4_ induced rat liver fibrosis.

Groups	LN (ng/ml)	HA (ng/ml)	IV-C (ng/ml)	PCIII (ng/ml)
Control	108.85 ± 8.56	88.83 ± 13.35	5.58 ± 0.36	10.41 ± 1.43
Model	142.65 ± 6.83^##^	120.61 ± 17.79^##^	7.48 ± 0.91^##^	14.41 ± 1.64^##^
AEPE	120.13 ± 8.44^**^	95.11 ± 13.14^**^	6.04 ± 1.16^*^	12.39 ± 1.54^*^
LWPE	112.90 ± 7.06^**^	96.42 ± 13.52^*^	6.01 ± 0.96^**^	12.47 ± 1.46^*^
HWPE	138.32 ± 12.18	118.76 ± 10.44	7.30 ± 0.27	14.26 ± 0.96
Silymarin	118.12 ± 11.71^**^	93.26 ± 16.95^**^	6.12 ± 1.05^*^	13.43 ± 1.24

Data were expressed as the mean ± SEM (n = 7–10). ^##^p < 0.01 vs the control group; *p < 0.05, **p < 0.01 vs the model group.

These results indicated that the anti-hepatic fibrosis active ingredients exist mainly in the low molecular weight fraction of the PE water extract, that is, the LWPE fraction. Therefore, the LWPE group was selected for in-depth proteomic analysis to elucidate the multitarget mechanism of PE against hepatic fibrosis.

### 3.4 Tandem mass tag-based proteomics analysis of liver tissue

**FIGURE 5:**
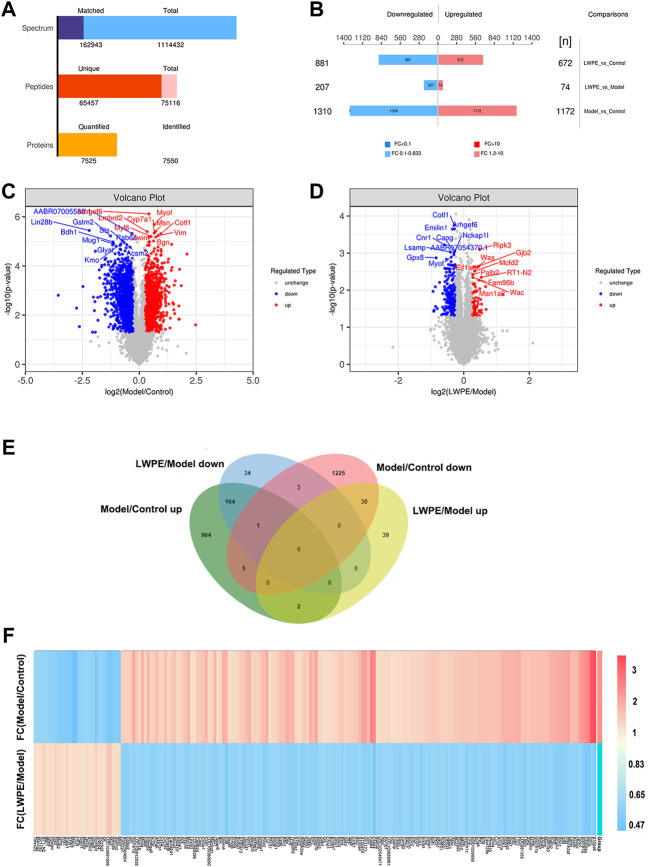
Identification of differentially expressed proteins (DEPs) in the livers of control, model and LWPE treated rats. **(A)** Basic statistics of the MS results of the samples. **(B)** The number of DEPs in different comparison groups. **(C** and **D)** Volcano plots of DEPs in different comparison groups. The red dots indicate upregulated proteins and the blue points show downregulated proteins screened based on fold change (FC) > 1.2 or <1/1.2 and a corrected *p*-value of <0.05. **(E)** Venn diagrams showing the distribution of overlapping proteins. **(F)** Hierarchical cluster analysis of 195 DEPs. The color indicates fold changes of proteins, dark blue indicates a decrease, while red indicates an increase.

A total of 65457 unique peptides and 7550 proteins were detected by TMT quantitative proteomics. Of these proteins, 7525 were quantified (Figure 5A). The relative changes in proteins are visualized in Figures 5B–D. Based on the cutoff value of a 1.2-fold change, in the Model/Control group, 1172 proteins were upregulated and 1310 proteins were downregulated (Supplementary Table S1). In the LWPE/Model group, 281 DEPs (74 upregulated and 207 downregulated) were identified. Importantly, 165 upregulated DEPs in the Model/Control group were downregulated in the LWPE/Model group, and 30 downregulated DEPs in the Model/Control group were upregulated in the LWPE/Model group (Figure 5E). The details of 195 DEPs that were reversely regulated by LWPE treatment are listed in Figure 5F; Supplementary Table S2.

### 3.5 Bioinformatics analysis of differentially expressed proteins

#### 3.5.1 Subcellular localization and domain analysis of differentially expressed proteins

**FIGURE 6:**
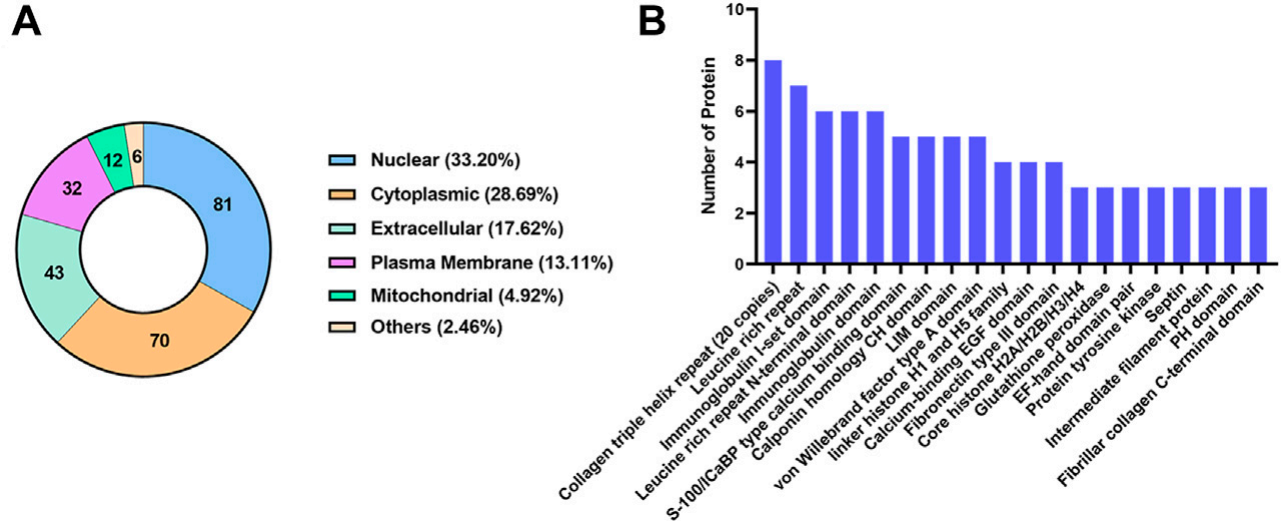
Classification of DEPs based on subcellular localization and domain. **(A)** The number and distribution ratio of DEPs in each subcellular organelle. **(B)** The number of proteins in the domains (top 20).

The subcellular localization analysis of DEPs is helpful to further comprehend the functions of the proteins in cells. CELLO software was applied to show the number and distribution ratio of DEPs in each subcellular organelle (Figure 6A). The DEPs were located mainly in the nucleus (33.20%), cytoplasmic (28.69%), extracellular (17.62%), and plasma membrane (13.11%). The domain prediction software InterProScan was used to predict the domains of DEPs, and the number of proteins in the domains (top 20) is shown (Figure 6B). The number of DEPs containing the collagen triple helix repeat (20 copies), leucine rich repeat, immunoglobulin I-set domain, leucine rich repeat N-terminal domain and immunoglobulin domain were the largest.

#### 3.5.2 Functional classification of differentially expressed proteins

**FIGURE 7:**
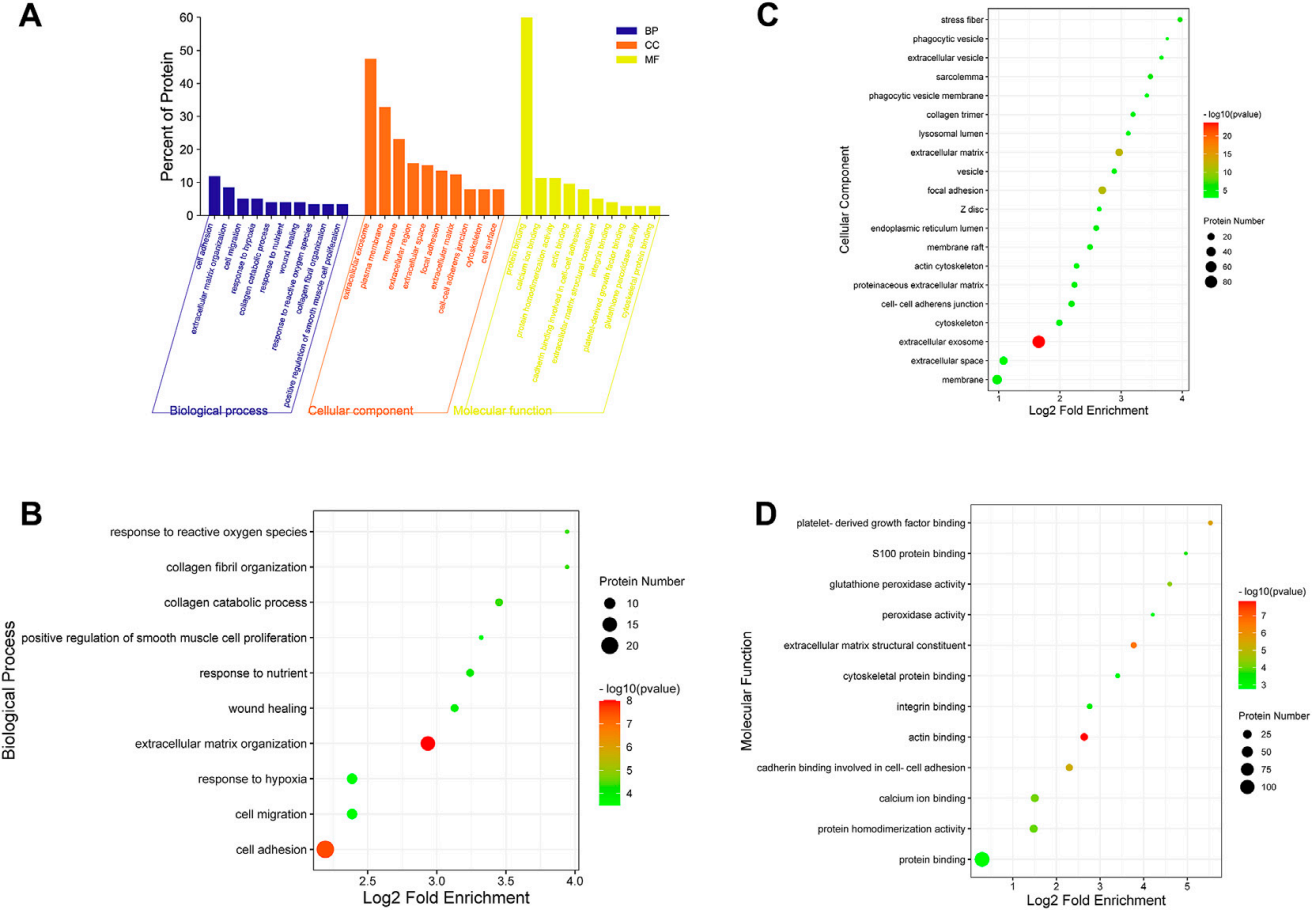
GO annotation classification analysis in biological process (BP), cellular component (CC) and molecular function (MF). **(A)** The top 10 significantly richer terms in the BP, CC, and MF categories. **(B)** Enrichment bubble plots of DEPs in BP. **(C)** Enrichment bubble plots of DEPs in the top 20 CC. **(D)** Enrichment bubble plots of DEPs in MF. *p* < 0.05, FDR<0.05.

For a comprehensive understanding of the function, localization and biological pathways of DEPs in living organisms, DEPs were annotated through GO analysis. Set *p*-value (*p*) < 0.05, false discovery rate (FDR) < 0.05, and a total of 52 items is obtained, of which 10 are BP, 12 are MF, and CC occupies 30, Figure 7A shows an overview of GO analysis, selecting top 10 significantly richer terms in the BP, CC, and MF categories, respectively.

For GO enrichment analysis, the results of BP, MF and the top 20 CC terms with the most significant enrichment are presented in bubble diagrams. In biological processes (Figure 7B), DEPs were primarily involved in extracellular matrix organization and cell adhesion. In molecular function, actin binding, extracellular matrix structural constituent, platelet-derived growth factor binding and protein binding were significantly regulated (Figure 7C). In the cellular component category, the results indicated that DEPs were significantly related to extracellular exosome, extracellular matrix and focal adhesion (Figure 7D).

#### 3.5.3 Kyoto Encyclopedia of Genes and Genomes pathway analysis of differentially expressed proteins

**FIGURE 8:**
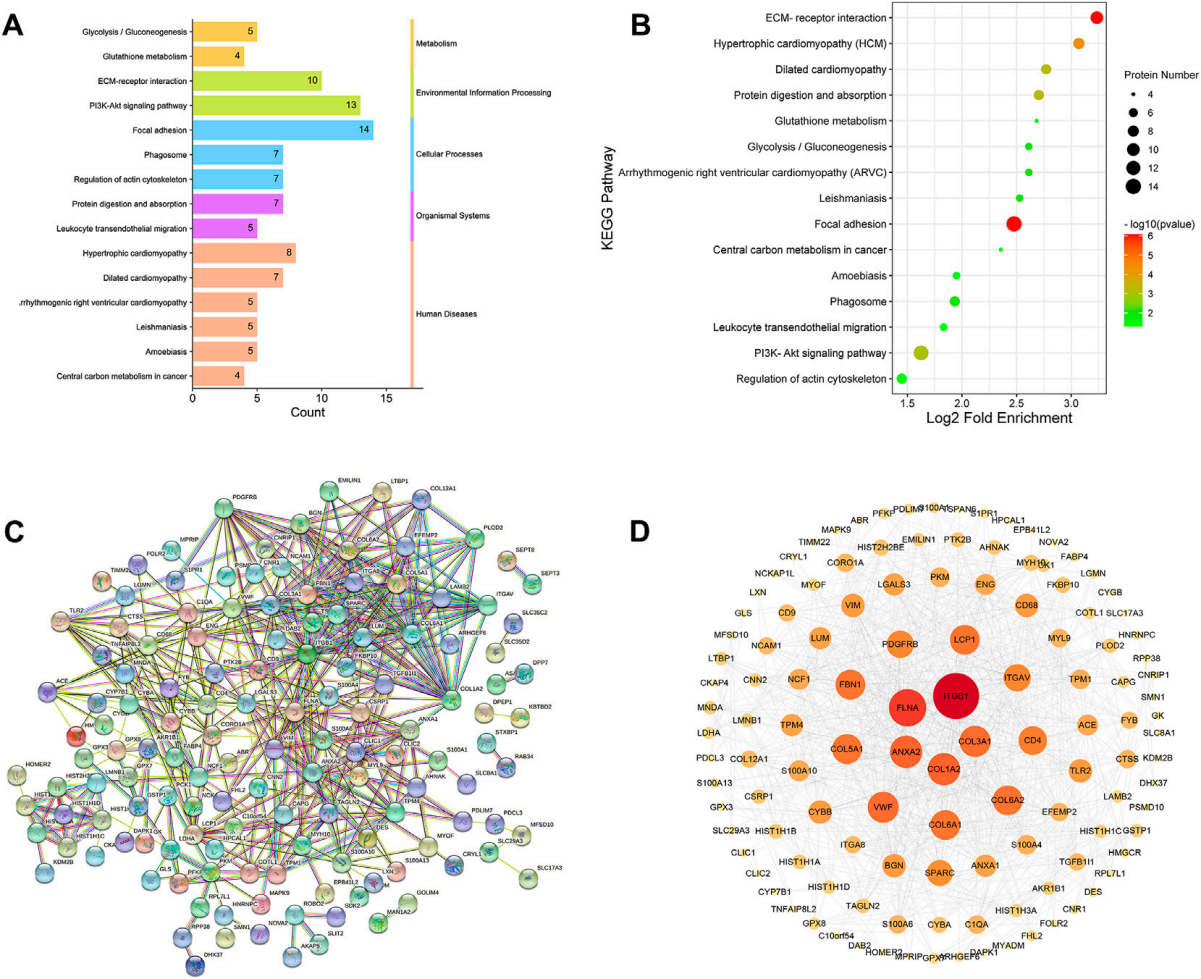
Kyoto Encyclopedia of Genes and Genomes (KEGG) pathway analysis and protein-protein interaction (PPI) network analysis of DEPs. **(A)** Classification of KEGG pathways. **(B)** Enrichment bubble plots of DEPs in the KEGG pathway, *p*<0.05. **(C)** PPI analysis among the DEPs of STRING database, medium confidence>0.4. **(D)** Cytoscape analysis of DEPs.

The pathway enrichment of DEPs was analyzed using a KEGG pathway analysis. Fifteen pathways were enriched (*p* <0.05, Supplementary Table S3). KEGG pathway enrichment bubble chart analyses revealed these DEPs to be enriched in ECM-receptor interaction, focal adhesion and PI3K-Akt signalling pathway (Figure 8A). To further explore the types of pathways enriched, the 15 pathways were classified as shown in Figure 8B, and most of these DEPs were enriched in human disease-related pathways, suggesting that the mechanisms underlying the antifibrotic effects of LWPE are multifunctional and involve multiple pathways.

### 3.5.4 Protein-protein interaction analysis of differentially expressed proteins

The STRING online database was used to determine the relationship among DEPs to elucidate the molecular mechanism underlying the crosstalk, and the protein interaction parameter score value was “medium confidence >0.4”. A total of 172 nodes and 456 edges were interconnected (Figure 8C). The PPI network was further constructed by Cytoscape software to analyze and visualize the importance of target proteins, and the results revealed that ITGB1, COL1A2, ITGAV, TLR2, ACE, and PDGFRB occupy the center of the PPI network and act as hubs for interaction with other differentially expressed proteins (Figure 8D).

### 3.6 Western blot analysis

**FIGURE 9:**
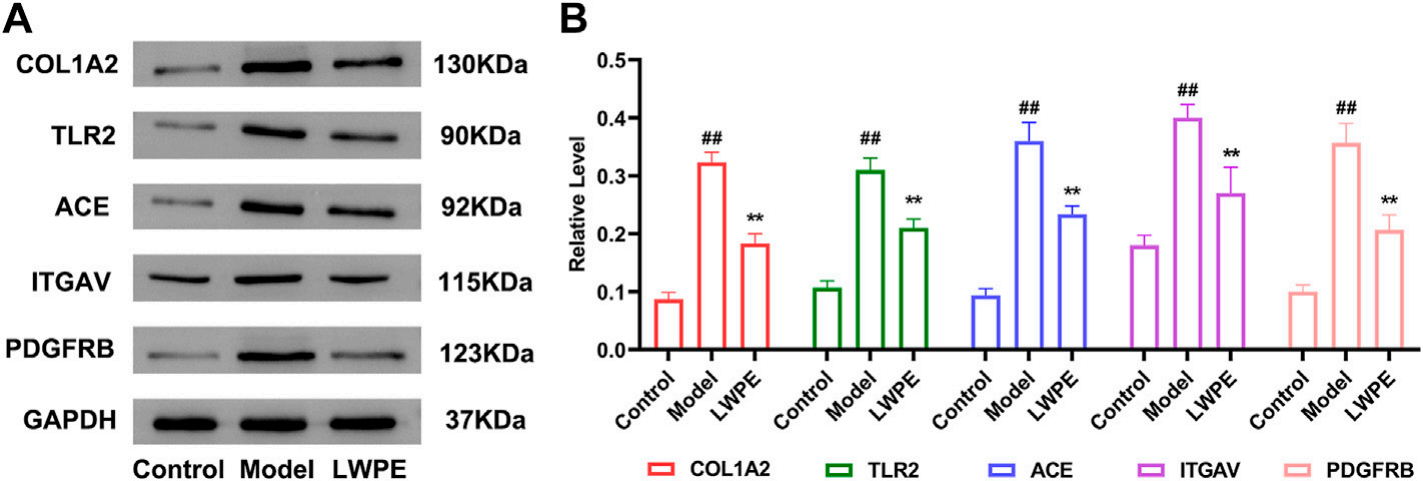
Western blot analysis of the key proteins. **(A)** The protein expression levels of GAPDH, COL1A2, ITGAV, TLR2, ACE and PDGFRB; **(B)** The relative levels of the proteins, in the model group vs the LWPE group. Data are expressed as the mean ± SEM (*n* = 3). ##*p* < 0.01 vs the control group; *p < 0.05, **p < 0.01 vs the model group.

The protein expression levels of COL1A2, ITGAV, TLR2, ACE, and PDGFRB in liver tissue were measured by Western blot to further verify the results of quantitative proteomics analysis. As shown in Figure 9, the expression levels of COL1A2, ITGAV, TLR2, ACE, and PDGFRB in the model group were all significantly increased compared with those in the control group (*p* < 0.01). LWPE treatment significantly downregulated the levels of these proteins compared with those in the model group (*p* < 0.01). The results are consistent with the protein profiles in TMT-based proteomic analysis.

## 4 Discussion

In the present study, the anti-hepatic fibrosis efficacies of AEPE and its fractions were evaluated using a rat model induced by CCl_4_. LWPE contained mainly secondary metabolites proven to be the optimal effective chemical fraction of AEPE by comprehensive serum biochemical analysis and histopathological examination. Our previous work demonstrated that LWPE contains a variety of phenolic acids and flavonoids, such as gallic acid, corilagin, ellagic acid, etc (Yin et al., 2021). These monomeric components have been reported to exert inhibitory effects on liver fibrosis by interfering with different molecular signalling pathways (Yin et al., 2022). Hence, LWPE possesses the characteristics of multicomponent and multitarget integrated therapy for liver fibrosis, which makes it difficult to elucidate the effective mechanism of LWPE.

Currently, high-throughput proteomic technology has been broadly applied to search for biomarkers and drug targets of liver diseases (Cowan et al., 2010), providing an appropriate technical tool for identifying the multitarget mechanism of LWPE on hepatic fibrosis. We identified 195 DEPs regulated by LWPE in liver tissue using TMT-based quantitative proteomics. Through GO enrichment analysis, the DEPs were highly related to ECM organization, ECM structural constituents and extracellular exosomes. During the formation of liver fibrosis, HSC are activated under the stimulation of various biological factors and then transformed into myofibroblasts, leading to excessive ECM deposition, which is a common pathological feature of liver fibrosis (Sun et al., 2018; Zhang et al., 2020). The ECM is composed of a heterogeneous mixture of glycoproteins and proteoglycans (PGs), including LN, fibronectin, collagen, HA and heparan sulfate PGs (Jonathan P. Myers et al., 2011). In our experimental pharmacodynamic results, the contents of HA, LN, IV-C, PCIII, and Hyp in the LWPE group were significantly decreased compared with those in the model group. Therefore, LWPE may alleviate CCl_4_-induced liver fibrosis by reducing ECM synthesis and accumulation.

KEGG pathway enrichment showed that DEPs are related to ECM-receptor interaction, focal adhesion and the PI3K-Akt signalling pathway. Interestingly, the ECM-receptor interaction and PI3K-Akt signalling pathways are involved in the focal adhesion (Chen et al., 2017). Studies have proven that focal adhesion plays an important role in HSC activation, and the disintegration of focal adhesions in activated HSCs may contribute to reversing liver fibrosis (Kumar et al., 2014). Besides, ECM is mainly distributed and aggregates on the cell surface and intercellular substances (Yumeng and Shenghua, 2017), and cell adhesion to ECM is mediated by ECM receptors namely integrins, discoidin domains and syndecans (Khurana et al., 2021). Integrins activate focal adhesion kinase (FAK) and Src-family kinases, and subsequently stimulate downstream signalling cascades such as the PI3K/Akt signalling pathway (Mitra et al., 2005). The expression of ECM in various cell types can be induced by activating the PI3K/Akt pathway (Sun et al., 2016). Inhibition of PI3K signalling in HSCs restrained collagen synthesis and ECM deposition, and decreased the expression of profibrogenic factors (Tu et al., 2016). Studies have shown that PE extract can inhibit Akt overactivity, thereby inhibiting the dysregulation of PI3K/Akt (Kunchana et al., 2021). We speculate that LWPE can alleviate liver fibrosis by regulating multiple pathways, including ECM-receptor interaction, focal adhesion and the PI3K-Akt signalling pathway.

In addition, according to the PPI network analysis, COL1A2, ITGAV, TLR2, ACE, and PDGFRB located in the center of the network and presented in multiple enriched signalling pathways. Therefore, these representative proteins were selected for further molecular biotechnology verification, and the results proved that LWPE can decrease the abovementioned proteins expression caused by CCl_4_.

Collagen is the most abundant ECM protein when fibrosis occurs, accounting for approximately 50% of the dry liver weight in cirrhosis (Kunchana et al., 2021). Among the 28 known types of collagen, at least 11 are expressed in liver tissue (Kunchana et al., 2021). In this study, quantitative protein analysis showed that various collagen proteins (including Col5a1, Col3a1, Col1a2, Col6a1, and Col6a2) in the liver tissue of model rats were decreased after treatment with LWPE. To date, there are no recognized most important proteins of the ECM specifically addressed in fibrosis, type I and III collagens are the most abundant collagens, followed by type IV, V, and VI collagens (Karsdal et al., 2015). Among these collagens, the type I collagen subunit molecule is a fibril-forming heterotrimeric protein consisting of two α1 chains and one α2 chain, which fold into a highly ordered and steady triple-helix (Blackstock et al., 2014). Col1a1 is a valid target for the treatment of liver fibrosis by inhibiting the synthesis of type I collagen, which has been proven in many studies (Calvente et al., 2015; Tao et al., 2018). To the best of our knowledge, the role of Col1a2 in liver fibrosis is still poorly understood. We validated that LWPE could reduce the expression of Col1a2 by Western blotting, suggesting that Col1a2 may be a potential effective target for the treatment of liver fibrosis.

Toll-like receptors (TLRs), a class of pattern recognition receptors, play a specific role in the regulation of the inflammatory response and liver fibrosis (Raby et al., 2017; Gan et al., 2018). TLR2 is one of the most common TLRs and is widely expressed on parenchymal and nonparenchymal liver cells mediating liver disease pathogenesis, including alcoholic liver disease and the nonalcoholic steatohepatitis (Getachew et al., 2021). TLR2 signalling pathways induce translocation of NF-κB into the nucleus and eventually modulate transcription of genes, as well as the production of inflammatory cytokines, considered to be the major hepatotoxic mediators that participate in the pathological process of liver fibrosis (Simeonova et al., 2001; Yang et al., 2020; Getachew et al., 2021). Knockout of TLR2 has been reported to be able to relieve CCl_4_-induced hepatic fibrosis in mice by downregulating the expression of profibrotic and proinflammatory genes (Ji et al., 2014). Previous studies have shown that various extracts and ingredients of PE possess anti-inflammatory properties (Li et al., 2020); hence, we speculated that TLR2 may be the key target that mediates the anti-inflammatory effects of LWPE in treating liver fibrosis. Moreover, the effects of LWPE on the TLR2/NF-κB signalling pathway in hepatic fibrosis rats also deserve further verification.

Inhibiting the activation and proliferation of HSCs in damaged livers has been widely recognized as a suitable treatment strategy for hepatic fibrosis (Yang et al., 2020). PDGF, which is the most potent mitogen for activated HSCs, binds to PDGF α and *ß* receptors and activates the downstream ERK/MAPK and Akt/PKB signalling pathways, leading to stimulation of HSCs proliferation (Borkham-Kamphorst and Weiskichen, 2016). However, only PDGF *ß* receptor (PDGFBR) is specifically overexpressed on activated HSCs (Chen et al., 2020). Reducing PDGFBR expression by siRNA effectively reduced the activation and proliferation of HSCs *in vitro* and suppressed liver fibrosis in an animal model (Chen et al., 2008). ITGAV, namely αV integrins, are heterodimeric cell-surface proteins, that play a central role in the progression of liver fibrosis as they activate latent TGF-β which is a known profibrogenic cytokine (Rahman et al., 2022). Experimental evidence demonstrates that depletion of the ITGAV subunit in HSCs protects mice from liver fibrosis induced by CCl_4_ (Henderson et al., 2013). ACE is a key proteolytic enzyme of the renin-angiotensin system (RAS), converting the decapeptide angiotensin I (Ang I) into the active octapeptide angiotensin II (Ang II) (Henderson et al., 2013). Ang II could induce contraction and proliferation of HSCs through the Ang II type 1 (AT1) receptor and motivate the activation and differentiation of quiescent HSCs into myofibroblasts (Saber et al., 2018). Several studies applied the method of ACE inhibition and showed that reducing Ang II formation could significantly ameliorate bile duct ligation or CCl_4_ induced liver fibrosis in rats (Simeonova et al., 2001). Therefore, we speculated that LWPE likely inhibited HSCs *via* interfering the expression of PDGFBR, ITGAV and ACE according to the results of proteomic analysis and Western blotting assays.

Taken together, these key DEPs are closely associated with the pathophysiology of hepatic fibrosis involving ECM sedimentation, inflammation, activation and proliferation of HSCs, which suggests that LWPE regulates liver fibrosis through multiple targets and multiple pathways. Additionally, many previous *in vivo* toxicity evaluation studies reported that PE extracts have no obvious toxic effects though hematological and histopathological examination, behavioral observation and biochemical marker analysis (Saini et al., 2022). Hence, PE is expected to be a potential source for the development of anti-liver fibrosis multitarget drugs that are safe and have no side effects.

## 5 Conclusion

In this study, we determined that LWPE is the main effective fraction of AEPE against liver fibrosis, and proteomics and bioinformatics analysis showed that LWPE can regulate multiple targets including COL1A2, ITGAV, TLR2, ACE, and PDGFRB, etc., ECM-receptor interaction, focal adhesion and the PI3K-Akt signalling pathway and other pathways to exert antifibrosis effects.

## Data Availability

The datasets presented in this study can be found in online repositories. The names of the repository/repositories and accession number(s) can be found in the article/Supplementary Material.

## Abbreviations

AEPE, the aqueous extract of PE; ALT, alanine aminotransferase; AST, aspartate aminotransferase; DEPs, differentially expressed proteins; ECM, extracellular matrix; ELISA, enzyme-linked immunosorbent assay; FC, fold change; GO, gene ontology; HA, hyaluronic acid; H&E, hematoxylin and eosin; HWPE, high molecular weight fraction of AEPE; HSC, hepatic stellate cell; Hyp, hydroxyproline; IV-C, collagen IV; KEGG, Kyoto Encyclopedia of Genes and Genomes; LF, liver fibrosis LN, lamininLWPE, low molecular weight fraction of AEPE; PCIII, type III precollagen; PDGF, platelet-derived growth factor; PE, phyllanthus emblica; TGF-β1, transforming growth factor-β1; TMT, tandem mass tag; WB, western blot.
