# Supplementary material for: Tandem mass tag-based proteomics analysis reveals the multitarget mechanisms of Phyllanthus emblica against liver fibrosis
Source: Front Pharmacol. 2022 Oct 13;13:989995. doi: 10.3389/fphar.2022.989995 (PMC9606415; doi:10.3389/fphar.2022.989995)
Supplement: Supplementary file 1 [file Table2.docx]

**Table S2. 195 DEPs regulated by LWPE.**

| Number | Accession | Protein name | Gene name | FC (Model/Control) | FC (LWPE/Model) | Regulation |
| --- | --- | --- | --- | --- | --- | --- |
| 1 | ENSRNOP00000067217 | histone cluster 1 H3 family member A | Hist1h3a | 4.273881486 | 0.665691398 | down |
| 2 | ENSRNOP00000015612 | S100 calcium binding protein A6 | S100a6 | 3.591081789 | 0.547905315 | down |
| 3 | ENSRNOP00000018634 | FYN binding protein 1 | Fyb1 | 3.403730313 | 0.69704138 | down |
| 4 | ENSRNOP00000013979 | glutathione peroxidase 8 | Gpx8 | 2.684382214 | 0.557096933 | down |
| 5 | ENSRNOP00000014701 | fatty acid binding protein 4 | Fabp4 | 2.646091749 | 0.829094976 | down |
| 6 | ENSRNOP00000010601 | cytoskeleton-associated protein 4 | Ckap4 | 2.573657852 | 0.53037831 | down |
| 7 | ENSRNOP00000063373 | coiled coil domain containing 88A | Ccdc88a | 2.475892261 | 0.570686281 | down |
| 8 | ENSRNOP00000023054 | histone cluster 1 H1 family member a | Hist1h1a | 2.436461739 | 0.66614961 | down |
| 9 | ENSRNOP00000047134 | tropomyosin 1 | Tpm1 | 2.246871186 | 0.800229231 | down |
| 10 | ENSRNOP00000008415 | olfactory receptor 1121 | Olr1121 | 2.197897637 | 0.695439653 | down |
| 11 | ENSRNOP00000022965 | histone cluster 1 H2A family member F | Hist1h2af | 2.189780214 | 0.690441488 | down |
| 12 | ENSRNOP00000037097 | S100 calcium binding protein A10 | S100a10 | 2.182528344 | 0.704084268 | down |
| 13 | ENSRNOP00000022029 | FK506 binding protein 10 | Fkbp10 | 2.139166432 | 0.739731225 | down |
| 14 | ENSRNOP00000021915 | Cd4 molecule | Cd4 | 2.117167002 | 0.684471634 | down |
| 15 | ENSRNOP00000007485 | ADP-ribosyltransferase 4 | Art4 | 2.092574832 | 0.625680125 | down |
| 16 | ENSRNOP00000065338 | histone cluster 1 H1 family member d | Hist1h1d | 2.066719738 | 0.746121325 | down |
| 17 | ENSRNOP00000042637 | interferon induced transmembrane protein 1 | Ifitm1 | 2.019945015 | 0.657454207 | down |
| 18 | ENSRNOP00000060365 | golgi integral membrane protein 4 | Golim4 | 2.017482604 | 0.813893251 | down |
| 19 | ENSRNOP00000026860 | desmin | Des | 2.008603064 | 0.680222623 | down |
| 20 | ENSRNOP00000048442 | neural cell adhesion molecule 1 | Ncam1 | 1.995500712 | 0.65439222 | down |
| 21 | ENSRNOP00000018402 | latexin | Lxn | 1.975082584 | 0.687114464 | down |
| 22 | ENSRNOP00000024030 | transgelin | Tagln | 1.960326866 | 0.743949101 | down |
| 23 | ENSRNOP00000023014 | four and a half LIM domains 2 | Fhl2 | 1.953496047 | 0.681291794 | down |
| 24 | ENSRNOP00000019121 | RAB32, member RAS oncogene family | Rab32 | 1.951957837 | 0.752063979 | down |
| 25 | ENSRNOP00000052876 | fibrillin 1 | Fbn1 | 1.946328096 | 0.704802918 | down |
| 26 | ENSRNOP00000014502 | lymphocyte cytosolic protein 1 | Lcp1 | 1.941942132 | 0.788000208 | down |
| 27 | ENSRNOP00000062744 | myosin heavy chain 10 | Myh10 | 1.935193515 | 0.73853511 | down |
| 28 | ENSRNOP00000013913 | glutathione peroxidase 7 | Gpx7 | 1.931868251 | 0.718014628 | down |
| 29 | ENSRNOP00000075258 | slit guidance ligand 2 | Slit2 | 1.928147288 | 0.713962222 | down |
| 30 | ENSRNOP00000017271 | dipeptidylpeptidase 7 | Dpp7 | 1.923513716 | 0.700956525 | down |
| 31 | ENSRNOP00000021954 | coactosin-like F-actin binding protein 1 | Cotl1 | 1.920657485 | 0.817868617 | down |
| 32 | ENSRNOP00000018442 | kelch repeat and BTB domain containing 2 | Kbtbd2 | 1.912306126 | 0.724775122 | down |
| 33 | ENSRNOP00000063646 | 3-oxoacid CoA transferase 1 | Oxct1 | 1.912178786 | 0.788941454 | down |
| 34 | ENSRNOP00000029762 | myeloid cell nuclear differentiation antigen | Mnda | 1.882696648 | 0.706739545 | down |
| 35 | ENSRNOP00000024601 | glutathione S-transferase pi 1 | Gstp1 | 1.882443235 | 0.647644811 | down |
| 36 | ENSRNOP00000026633 | CD9 molecule | Cd9 | 1.875285401 | 0.598517993 | down |
| 37 | ENSRNOP00000026496 | coronin 1A | Coro1a | 1.868114599 | 0.793657375 | down |
| 38 | ENSRNOP00000072344 | EGF containing fibulin extracellular matrix protein 2 | Efemp2 | 1.859045183 | 0.80571242 | down |
| 39 | ENSRNOP00000015958 | S100 calcium-binding protein A4 | S100a4 | 1.858961495 | 0.825609328 | down |
| 40 | ENSRNOP00000028642 | TNF alpha induced protein 8 like 2 | Tnfaip8l2 | 1.85506355 | 0.735794503 | down |
| 41 | ENSRNOP00000062926 | OTU deubiquitinase with linear linkage specificity like | Otulinl | 1.836042361 | 0.701237313 | down |
| 42 | ENSRNOP00000027445 | myosin light chain 9 | Myl9 | 1.823652579 | 0.74197725 | down |
| 43 | ENSRNOP00000047828 | procollagen lysine, 2-oxoglutarate 5-dioxygenase 2 | Plod2 | 1.821339987 | 0.66769767 | down |
| 44 | ENSRNOP00000058151 | protein tyrosine kinase 7 | Ptk7 | 1.813829395 | 0.700894503 | down |
| 45 | ENSRNOP00000063893 | septin 8 | Sept8 | 1.795098678 | 0.735397401 | down |
| 46 | ENSRNOP00000010101 | legumain | Lgmn | 1.791038838 | 0.749184229 | down |
| 47 | ENSRNOP00000033018 | TBC1 domain family member 1 | Tbc1d1 | 1.787624496 | 0.766069072 | down |
| 48 | ENSRNOP00000024430 | vimentin | Vim | 1.773165033 | 0.748598815 | down |
| 49 | ENSRNOP00000069212 | aldehyde dehydrogenase 1 family, member A3 | Aldh1a3 | 1.766633002 | 0.748684318 | down |
| 50 | ENSRNOP00000045501 | myosin phosphatase Rho interacting protein | Mprip | 1.760966938 | 0.717854268 | down |
| 51 | ENSRNOP00000038428 | annexin A2 | Anxa2 | 1.755344518 | 0.794468551 | down |
| 52 | ENSRNOP00000054774 | NOVA alternative splicing regulator 2 | Nova2 | 1.751842093 | 0.709959831 | down |
| 53 | ENSRNOP00000018899 | PDZ and LIM domain 7 | Pdlim7 | 1.750052884 | 0.734952953 | down |
| 54 | ENSRNOP00000015210 | raftlin lipid raft linker 1 | Rftn1 | 1.747709462 | 0.775989936 | down |
| 55 | ENSRNOP00000074300 | histone cluster 1 H1 family member b | Hist1h1b | 1.744875812 | 0.785681832 | down |
| 56 | ENSRNOP00000016067 | cytoglobin | Cygb | 1.742326171 | 0.702155993 | down |
| 57 | ENSRNOP00000069026 | AHNAK nucleoprotein | Ahnak | 1.742238643 | 0.763844198 | down |
| 58 | ENSRNOP00000016423 | collagen type I alpha 2 chain | Col1a2 | 1.737083232 | 0.700693288 | down |
| 59 | ENSRNOP00000068464 | proline and arginine rich end leucine rich repeat protein | Prelp | 1.718929932 | 0.752405397 | down |
| 60 | ENSRNOP00000074552 | myeloid-associated differentiation marker | Myadm | 1.71842113 | 0.716326519 | down |
| 61 | ENSRNOP00000072748 | axin interactor, dorsalization associated | Aida | 1.704591471 | 0.799273693 | down |
| 62 | ENSRNOP00000054664 | ribonuclease P/MRP subunit p38 | Rpp38 | 1.700888951 | 0.68675475 | down |
| 63 | ENSRNOP00000040099 | latent transforming growth factor beta binding protein 1 | Ltbp1 | 1.697219977 | 0.641541335 | down |
| 64 | ENSRNOP00000056498 | similar to potassium channel tetramerisation domain containing 12b | LOC681355 | 1.686163931 | 0.76726785 | down |
| 65 | ENSRNOP00000023664 | annexin A1 | Anxa1 | 1.676705317 | 0.757158142 | down |
| 66 | ENSRNOP00000006109 | lumican | Lum | 1.67636695 | 0.781081405 | down |
| 67 | ENSRNOP00000021073 | tropomyosin 4 | Tpm4 | 1.67621344 | 0.796284234 | down |
| 68 | ENSRNOP00000071060 | proteasome 26S subunit, non-ATPase 10 | Psmd10 | 1.673940596 | 0.766377121 | down |
| 69 | ENSRNOP00000004956 | collagen type III alpha 1 chain | Col3a1 | 1.672324539 | 0.752139847 | down |
| 70 | ENSRNOP00000067293 | glucosamine-6-phosphate deaminase 1 | Gnpda1 | 1.667234224 | 0.765930293 | down |
| 71 | ENSRNOP00000021689 | family with sequence similarity 129, member B | Fam129b | 1.656192368 | 0.744088533 | down |
| 72 | ENSRNOP00000066355 | endoglin | Eng | 1.655498375 | 0.725188886 | down |
| 73 | ENSRNOP00000072449 | biglycan | Bgn | 1.653304676 | 0.740912117 | down |
| 74 | ENSRNOP00000017385 | complement C1q A chain | C1qa | 1.652789621 | 0.820494452 | down |
| 75 | ENSRNOP00000026754 | HDGF like 3 | Hdgfl3 | 1.648811678 | 0.681078423 | down |
| 76 | ENSRNOP00000074052 | four and a half LIM domains 1 | Fhl1 | 1.648281482 | 0.718198557 | down |
| 77 | ENSRNOP00000067453 | centrin-3-like | LOC100912538 | 1.629339565 | 0.795885806 | down |
| 78 | ENSRNOP00000025512 | similar to RIKEN cDNA 2310022B05 | RGD1559896 | 1.618561023 | 0.75691002 | down |
| 79 | ENSRNOP00000070703 | filamin A | Flna | 1.603340881 | 0.810658845 | down |
| 80 | ENSRNOP00000073681 | histone cluster 1 H1 family member c | Hist1h1c | 1.599074219 | 0.671498479 | down |
| 81 | ENSRNOP00000031615 | protein tyrosine kinase 2 beta | Ptk2b | 1.598152695 | 0.814860245 | down |
| 82 | ENSRNOP00000031063 | cytochrome b-245 beta chain | Cybb | 1.598018889 | 0.825991016 | down |
| 83 | ENSRNOP00000017386 | major facilitator superfamily domain containing 10 | Mfsd10 | 1.595460676 | 0.796815768 | down |
| 84 | ENSRNOP00000023252 | phosphofructokinase, platelet | Pfkp | 1.594984762 | 0.777041114 | down |
| 85 | ENSRNOP00000011991 | cysteine and glycine-rich protein 1 | Csrp1 | 1.585075124 | 0.783265052 | down |
| 86 | ENSRNOP00000000689 | solute carrier family 29 member 3 | Slc29a3 | 1.578619201 | 0.80373518 | down |
| 87 | ENSRNOP00000021546 | myoferlin | Myof | 1.577462956 | 0.767829922 | down |
| 88 | ENSRNOP00000010627 | angiotensin I converting enzyme | Ace | 1.571956513 | 0.795362176 | down |
| 89 | ENSRNOP00000017486 | secreted protein acidic and cysteine rich | Sparc | 1.570580163 | 0.789496059 | down |
| 90 | ENSRNOP00000069474 | pyruvate kinase M1/2 | Pkm | 1.570101001 | 0.734048264 | down |
| 91 | ENSRNOP00000060240 | chloride intracellular channel 1 | Clic1 | 1.556173451 | 0.815829007 | down |
| 92 | ENSRNOP00000017882 | phosducin-like 3 | Pdcl3 | 1.55539149 | 0.818136026 | down |
| 93 | ENSRNOP00000016519 | S100 calcium binding protein A13 | S100a13 | 1.552703558 | 0.801654397 | down |
| 94 | ENSRNOP00000010491 | septin 3 | Sept3 | 1.549838324 | 0.703125958 | down |
| 95 | ENSRNOP00000018562 | capping actin protein, gelsolin like | Capg | 1.546356339 | 0.825930288 | down |
| 96 | ENSRNOP00000015559 | calcium/calmodulin-dependent protein kinase II delta | Camk2d | 1.544337045 | 0.827810519 | down |
| 97 | ENSRNOP00000002028 | neutrophil cytosolic factor 1 | Ncf1 | 1.541087557 | 0.813941616 | down |
| 98 | ENSRNOP00000021397 | dipeptidase 1 | Dpep1 | 1.533532855 | 0.759897908 | down |
| 99 | ENSRNOP00000028732 | cathepsin S | Ctss | 1.531378013 | 0.805704307 | down |
| 100 | ENSRNOP00000075422 | collagen type XII alpha 1 chain | Col12a1 | 1.524642448 | 0.758274451 | down |
| 101 | ENSRNOP00000071246 | ABR, RhoGEF and GTPase activating protein | Abr | 1.519479541 | 0.823686657 | down |
| 102 | ENSRNOP00000063042 | calponin 2 | Cnn2 | 1.514786119 | 0.721334484 | down |
| 103 | ENSRNOP00000006961 | integrin subunit alpha V | Itgav | 1.508915368 | 0.724504317 | down |
| 104 | ENSRNOP00000074171 | calcium voltage-gated channel auxiliary subunit alpha2delta 1 | Cacna2d1 | 1.496760196 | 0.767552578 | down |
| 105 | ENSRNOP00000070203 | galectin 3 | Lgals3 | 1.496175281 | 0.822074287 | down |
| 106 | ENSRNOP00000022713 | integrin subunit alpha 8 | Itga8 | 1.483751445 | 0.770533189 | down |
| 107 | ENSRNOP00000072215 | similar to LOC387763 protein | RGD1564664 | 1.477687169 | 0.669590177 | down |
| 108 | ENSRNOP00000017564 | cytochrome b-245 alpha chain | Cyba | 1.475413253 | 0.816748403 | down |
| 109 | ENSRNOP00000073111 | cytochrome P450 family 7 subfamily B member 1 | Cyp7b1 | 1.473994209 | 0.782033609 | down |
| 110 | ENSRNOP00000027009 | protocadherin gamma subfamily A, 8 | Pcdhga8 | 1.472055594 | 0.729199911 | down |
| 111 | ENSRNOP00000012334 | collagen type V alpha 1 chain | Col5a1 | 1.469228707 | 0.778244539 | down |
| 112 | ENSRNOP00000024693 | BOK, BCL2 family apoptosis regulator | Bok | 1.466180293 | 0.82027423 | down |
| 113 | ENSRNOP00000013025 | toll-like receptor 2 | Tlr2 | 1.458576844 | 0.76757802 | down |
| 114 | ENSRNOP00000022055 | 3-hydroxy-3-methylglutaryl-CoA reductase | Hmgcr | 1.455929442 | 0.815274938 | down |
| 115 | ENSRNOP00000060534 | platelet derived growth factor receptor beta | Pdgfrb | 1.450836741 | 0.776102329 | down |
| 116 | ENSRNOP00000001695 | collagen type VI alpha 2 chain | Col6a2 | 1.439005461 | 0.710004215 | down |
| 117 | ENSRNOP00000062573 | sidekick cell adhesion molecule 2 | Sdk2 | 1.435066606 | 0.809094016 | down |
| 118 | ENSRNOP00000051863 | transforming growth factor beta 1 induced transcript 1 | Tgfb1i1 | 1.433170126 | 0.822583859 | down |
| 119 | ENSRNOP00000013463 | N-acylsphingosine amidohydrolase 1 | Asah1 | 1.425304493 | 0.80812788 | down |
| 120 | ENSRNOP00000058936 | NCK associated protein 1 like | Nckap1l | 1.425302402 | 0.811587286 | down |
| 121 | ENSRNOP00000007816 | glutamate-rich 5 | Erich5 | 1.421344394 | 0.750059456 | down |
| 122 | ENSRNOP00000010850 | cannabinoid receptor 1 | Cnr1 | 1.420090816 | 0.665144164 | down |
| 123 | ENSRNOP00000014152 | nuclear receptor subfamily 2, group F, member 2 | Nr2f2 | 1.416728964 | 0.786503936 | down |
| 124 | ENSRNOP00000012663 | MARCKS-like 1 | Marcksl1 | 1.410729386 | 0.761471852 | down |
| 125 | ENSRNOP00000009328 | epidermal growth factor receptor pathway substrate 8 | Eps8 | 1.410280233 | 0.803894658 | down |
| 126 | ENSRNOP00000042464 | histone cluster 2 H2B family member E | Hist2h2be | 1.408606495 | 0.669234841 | down |
| 127 | ENSRNOP00000040765 | solute carrier family 8 member A1 | Slc8a1 | 1.408342442 | 0.813224954 | down |
| 128 | ENSRNOP00000021178 | syntaxin binding protein 1 | Stxbp1 | 1.403833552 | 0.82824922 | down |
| 129 | ENSRNOP00000053756 | Cd68 molecule | Cd68 | 1.403695627 | 0.780579247 | down |
| 130 | ENSRNOP00000026643 | von Willebrand factor | Vwf | 1.402876662 | 0.80479081 | down |
| 131 | ENSRNOP00000057979 | ATP-binding cassette, subfamily G (WHITE), member 3-like 3 | Abcg3l3 | 1.394453143 | 0.769722536 | down |
| 132 | ENSRNOP00000026925 | folate receptor beta | Folr2 | 1.380314248 | 0.69237368 | down |
| 133 | ENSRNOP00000030626 | transforming, acidic coiled-coil containing protein 1 | Tacc1 | 1.379083234 | 0.787913707 | down |
| 134 | ENSRNOP00000010932 | elastin microfibril interfacer 1 | Emilin1 | 1.374779168 | 0.785900573 | down |
| 135 | ENSRNOP00000028416 | neurotrophin receptor associated death domain | Nradd | 1.35930422 | 0.735325751 | down |
| 136 | ENSRNOP00000027375 | regulator of G-protein signaling 10 | Rgs10 | 1.353635418 | 0.761370813 | down |
| 137 | ENSRNOP00000024456 | survival of motor neuron 1, telomeric | Smn1 | 1.348933236 | 0.784782044 | down |
| 138 | ENSRNOP00000065052 | laminin subunit beta 2 | Lamb2 | 1.347316071 | 0.797432639 | down |
| 139 | ENSRNOP00000001158 | Rac/Cdc42 guanine nucleotide exchange factor 6 | Arhgef6 | 1.346384173 | 0.821341568 | down |
| 140 | ENSRNOP00000017000 | S100 calcium binding protein A1 | S100a1 | 1.345367131 | 0.693260383 | down |
| 141 | ENSRNOP00000043878 | DAB2, clathrin adaptor protein | Dab2 | 1.341959579 | 0.81781447 | down |
| 142 | ENSRNOP00000070487 | selenoprotein W-like | LOC103689961 | 1.330050431 | 0.808950042 | down |
| 143 | ENSRNOP00000009706 | RNA binding motif protein 18 | Rbm18 | 1.328707437 | 0.814196277 | down |
| 144 | ENSRNOP00000007374 | hippocalcin-like 1 | Hpcal1 | 1.328102822 | 0.821131965 | down |
| 145 | ENSRNOP00000012879 | aldo-keto reductase family 1 member B | Akr1b1 | 1.325538168 | 0.805166547 | down |
| 146 | ENSRNOP00000014785 | integrin subunit beta 1 | Itgb1 | 1.325261588 | 0.796561104 | down |
| 147 | ENSRNOP00000070966 | roundabout guidance receptor 2 | Robo2 | 1.322126195 | 0.820753952 | down |
| 148 | ENSRNOP00000065443 | tear acid-lipase-like protein-like | LOC100360690 | 1.316436042 | 0.786156398 | down |
| 149 | ENSRNOP00000058049 | erythrocyte membrane protein band 4.1-like 2 | Epb41l2 | 1.310547207 | 0.830272193 | down |
| 150 | ENSRNOP00000047354 | short transient receptor potential channel 2-like | LOC102549471 | 1.293409207 | 0.750473852 | down |
| 151 | ENSRNOP00000076046 | FERM domain containing 8 | Frmd8 | 1.288809124 | 0.794070392 | down |
| 152 | ENSRNOP00000036566 | RAB34, member RAS oncogene family | Rab34 | 1.280295987 | 0.820363115 | down |
| 153 | ENSRNOP00000068253 | septin 4 | Sept4 | 1.277011973 | 0.79726328 | down |
| 154 | ENSRNOP00000049916 | similar to RNA polymerase II elongation factor ELL2 | LOC689396 | 1.273616521 | 0.764879042 | down |
| 155 | ENSRNOP00000021803 | ribosomal protein L7-like 1 | Rpl7l1 | 1.273499633 | 0.832064037 | down |
| 156 | ENSRNOP00000073271 | glutathione peroxidase 3 | Gpx3 | 1.270757637 | 0.720177088 | down |
| 157 | ENSRNOP00000007104 | cannabinoid receptor interacting protein 1 | Cnrip1 | 1.258011528 | 0.786322169 | down |
| 158 | ENSRNOP00000001679 | collagen type VI alpha 1 chain | Col6a1 | 1.25556274 | 0.78534495 | down |
| 159 | ENSRNOP00000000955 | chloride intracellular channel 2 | Clic2 | 1.250444357 | 0.827390403 | down |
| 160 | ENSRNOP00000071972 | glutaminase | Gls | 1.243986859 | 0.832553375 | down |
| 161 | ENSRNOP00000019351 | lamin B1 | Lmnb1 | 1.240121426 | 0.832638074 | down |
| 162 | ENSRNOP00000047121 | limbic system-associated membrane protein | Lsamp | 1.234547972 | 0.753709932 | down |
| 163 | ENSRNOP00000054563 | V-set immunoregulatory receptor | Vsir | 1.231772653 | 0.801260658 | down |
| 164 | ENSRNOP00000005165 | tetraspanin 6 | Tspan6 | 1.216046272 | 0.78293558 | down |
| 165 | ENSRNOP00000038631 | DEAH-box helicase 37 | Dhx37 | 1.214218729 | 0.81142912 | down |
| 166 | ENSRNOP00000010779 | translocase of inner mitochondrial membrane 22 | Timm22 | 0.831092999 | 1.242130329 | up |
| 167 | ENSRNOP00000030747 | torsin family 2, member A | Tor2a | 0.824426959 | 1.273414035 | up |
| 168 | ENSRNOP00000060243 | mannosidase, alpha, class 1A, member 2 | Man1a2 | 0.8200888 | 1.211635556 | up |
| 169 | ENSRNOP00000070109 | solute carrier family 17 member 3 | Slc17a3 | 0.79229656 | 1.216399571 | up |
| 170 | ENSRNOP00000049883 | RT1 class Ib, locus N2 | RT1-N2 | 0.781967689 | 1.448842276 | up |
| 171 | ENSRNOP00000003987 | mitogen-activated protein kinase 9 | Mapk9 | 0.781267204 | 1.246016732 | up |
| 172 | ENSRNOP00000040894 | mitochondrial fission regulator 1 | Mtfr1 | 0.767745586 | 1.274506385 | up |
| 173 | ENSRNOP00000038617 | solute carrier family 35 member D2 | Slc35d2 | 0.766472024 | 1.267218026 | up |
| 174 | ENSRNOP00000072951 | solute carrier family 35 member C2 | Slc35c2 | 0.742695774 | 1.203743642 | up |
| 175 | ENSRNOP00000008416 | A-kinase anchoring protein 5 | Akap5 | 0.740949995 | 1.227218793 | up |
| 176 | ENSRNOP00000001432 | ADP-ribosylation factor like GTPase 6 interacting protein 4 | Arl6ip4 | 0.738138546 | 1.240188524 | up |
| 177 | ENSRNOP00000001912 | GRB10 interacting GYF protein 1 | Gigyf1 | 0.734022836 | 1.200191152 | up |
| 178 | ENSRNOP00000017468 | lactate dehydrogenase A | Ldha | 0.723600199 | 1.212263845 | up |
| 179 | ENSRNOP00000020233 | insulin-like growth factor binding protein, acid labile subunit | Igfals | 0.717116439 | 1.275621973 | up |
| 180 | ENSRNOP00000070427 | heterogeneous nuclear ribonucleoprotein C | Hnrnpc | 0.715789171 | 1.264258015 | up |
| 181 | ENSRNOP00000011711 | gap junction protein, beta 2 | Gjb2 | 0.687637478 | 1.373070104 | up |
| 182 | ENSRNOP00000059825 | glycerol kinase | Gk | 0.669668919 | 1.208973771 | up |
| 183 | ENSRNOP00000056394 | aldehyde dehydrogenase 1 family, member L2 | Aldh1l2 | 0.636053232 | 1.302590533 | up |
| 184 | ENSRNOP00000069073 | molybdenum cofactor synthesis 2 | Mocs2 | 0.618958137 | 1.364260678 | up |
| 185 | ENSRNOP00000018892 | oligodendrocyte-myelin glycoprotein | Omg | 0.610427462 | 1.318933951 | up |
| 186 | ENSRNOP00000030913 | phosphoenolpyruvate carboxykinase 1 | Pck1 | 0.607267993 | 1.229487005 | up |
| 187 | ENSRNOP00000062122 | death associated protein kinase 1 | Dapk1 | 0.60215332 | 1.401620285 | up |
| 188 | ENSRNOP00000066295 | mitochondrial translational initiation factor 3 | Mtif3 | 0.601792587 | 1.293111353 | up |
| 189 | ENSRNOP00000050408 | lysine demethylase 2B | Kdm2b | 0.597408096 | 1.271557608 | up |
| 190 | ENSRNOP00000052627 | sphingosine-1-phosphate receptor 1 | S1pr1 | 0.592496987 | 1.246738866 | up |
| 191 | ENSRNOP00000070858 | homer scaffold protein 2 | Homer2 | 0.566300269 | 1.447277446 | up |
| 192 | ENSRNOP00000076305 | uncharacterized LOC103691699 | LOC103691699 | 0.549473325 | 1.605207637 | up |
| 193 | ENSRNOP00000006995 | melanoma inhibitory activity 2 | Mia2 | 0.539179319 | 1.589388731 | up |
| 194 | ENSRNOP00000013553 | MAGI family member, X-linked | Magix | 0.48758348 | 1.224204722 | up |
| 195 | ENSRNOP00000012093 | crystallin, lambda 1 | Cryl1 | 0.472928971 | 1.286842498 | up |
