# Supplementary material for: Tandem mass tag-based proteomics analysis reveals the multitarget mechanisms of Phyllanthus emblica against liver fibrosis
Source: Front Pharmacol. 2022 Oct 13;13:989995. doi: 10.3389/fphar.2022.989995 (PMC9606415; doi:10.3389/fphar.2022.989995)
Supplement: Supplementary file 2 [file Table3.docx]

**Table S3. KEGG pathway enrichment analysis of 195 DEPs.**

| Term | Count | P Value | Genes | FDR |
| --- | --- | --- | --- | --- |
| hsa04512: ECM-receptor interaction | 10 | 8.33E-07 | ITGB1, COL3A1, COL1A2, COL5A1, VWF, LAMB2, COL6A2, COL6A1, ITGA8, ITGAV | 7.30E-05 |
| hsa04510: Focal adhesion | 14 | 8.90E-07 | PDGFRB, ITGB1, VWF, LAMB2, MAPK9, COL3A1, COL1A2, COL5A1, COL6A2, COL6A1, ITGA8, FLNA, ITGAV, MYL9 | 7.30E-05 |
| hsa05410: Hypertrophic cardiomyopathy (HCM) | 8 | 3.82E-05 | ITGB1, ACE, DES, TPM4, CACNA2D1, TPM1, ITGA8, ITGAV | 0.002086819 |
| hsa05414: Dilated cardiomyopathy | 7 | 6.33E-04 | COL3A1, COL1A2, COL5A1, COL6A2, COL12A1, COL6A1, SLC8A1 | 0.020768414 |
| hsa04974: Protein digestion and absorption | 7 | 6.33E-04 | COL3A1, COL1A2, COL5A1, COL6A2, COL12A1, COL6A1, SLC8A1 | 0.020768414 |
| hsa04151: PI3K-Akt signaling pathway | 13 | 8.08E-04 | PDGFRB, ITGB1, VWF, LAMB2, COL3A1, COL1A2, COL5A1, COL6A2, COL6A1, ITGA8, ITGAV, PCK1, TLR2 | 0.022081568 |
| hsa05412: Arrhythmogenic right ventricular cardiomyopathy (ARVC) | 5 | 0.008488193 | ITGB1, DES, CACNA2D1, ITGA8, ITGAV | 0.168529544 |
| hsa00010: Glycolysis / Gluconeogenesis | 5 | 0.008488193 | ALDH1A3, LDHA, PKM, PCK1, PFKP | 0.168529544 |
| hsa04145: Phagosome | 7 | 0.009248573 | ITGB1, NCF1, ITGAV, CYBA, CORO1A, CTSS, TLR2 | 0.168529544 |
| hsa05140: Leishmaniasis | 5 | 0.010377291 | ITGB1, MARCKSL1, NCF1, CYBA, TLR2 | 0.170187572 |
| hsa00480: Glutathione metabolism | 4 | 0.02328098 | GPX3, GSTP1, GPX8, GPX7 | 0.347098254 |
| hsa05146: Amoebiasis | 5 | 0.038727205 | COL3A1, COL1A2, COL5A1, LAMB2, TLR2 | 0.488405296 |
| hsa04810: Regulation of actin cytoskeleton | 7 | 0.040683507 | PDGFRB, ITGB1, ITGA8, ITGAV, NCKAP1L, MYL9, ARHGEF6 | 0.488405296 |
| hsa05230: Central carbon metabolism in cancer | 4 | 0.041693135 | PDGFRB, PKM, PFKP, GLS | 0.488405296 |
| hsa04670: Leukocyte transendothelial migration | 5 | 0.049737392 | ITGB1, NCF1, PTK2B, CYBA, MYL9 | 0.54379549 |
